# Natural history and prognostic implications of left ventricular end-diastolic pressure in reperfused ST-segment elevation myocardial infarction: an analysis of the thrombolysis in myocardial infarction (TIMI) II randomized controlled trial

**DOI:** 10.1186/s12872-021-02046-x

**Published:** 2021-05-17

**Authors:** Arshad A. Khan, Mohammed S. Al-Omary, Nicholas J. Collins, John Attia, Andrew J. Boyle

**Affiliations:** 1 Department of Cardiovascular Medicine, John Hunter Hospital, Locked Bag 1, HRMC, Newcastle, NSW 2310 Australia; 2 The University of Newcastle, Newcastle, Australia; 3 Hunter Medical Research Institute, Newcastle, Australia

**Keywords:** Left ventricular end diastolic pressure, ST-segment elevation myocardial infarction

## Abstract

**Background:**

The aim of the current study is to assess the natural history and prognostic value of elevated left ventricular end-diastolic pressure (LVEDP) in patients with ST-segment elevation myocardial infarction (STEMI) after reperfusion with thrombolysis; we utilize data from the Thrombolysis in Myocardial Infarction (TIMI) II study.

**Methods:**

A total of 3339 patients were randomized to either an invasive (n = 1681) or a conservative (n = 1658) strategy in the TIMI II study following thrombolysis. To make the current cohort as relevant as possible to modern pharmaco-invasively managed cohorts, patients in the invasive arm with TIMI flow grade ≥ 2 (N = 1201) at initial catheterization are included in the analysis. Of these, 259 patients had a second catheterization prior to hospital discharge, and these were used to define the natural history of LVEDP in reperfused STEMI.

**Results:**

The median LVEDP for the whole cohort was 18 mmHg (IQR: 12–23). Patients were divided into quartiles by LVEDP measured during the first cardiac catheterization. During a median follow up of 3 (IQR: 2.1–3.2) years, quartile 4 (highest LVEDP) had the highest incidence of mortality and heart failure admissions. In the cohort with paired catheterization data, the LVEDP dropped slightly from 18 mmHg (1QR: 12–22) to 15 mmHg (IQR: 10–20) (p = 0.01) from the first to the pre-hospital discharge catheterization.

**Conclusions:**

LVEDP remains largely stable during hospitalisation post-STEMI. Elevated LVEDP is a predictor of death and heart failure hospitalization in STEMI patients undergoing successful thrombolysis.

**Graphic abstract:**

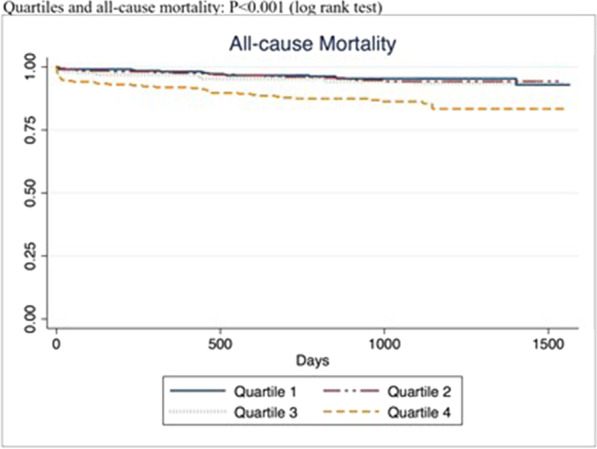

## Background

ST-segment elevation myocardial infarction (STEMI) affects both left ventricular systolic and diastolic function [1]. Although the prognostic utility of reduced left ventricular ejection fraction (LVEF) [2, 3] along with patients’ age, infarct size and location, ventricular arrhythmias, ischemic mitral regurgitation, and cardiogenic shock are well established, the prognostic implications of diastolic indices in STEMI have rarely been explored, despite diastolic dysfunction either preceding or occurring independent of systolic dysfunction [4–6]. Left ventricular end-diastolic pressure (LVEDP), which reflects global left ventricular compliance [7], is easily measured during cardiac catheterization. However, no studies have evaluated the natural history of LVEDP after reperfusion of the infarct artery, and few studies have assessed its usefulness in predicting outcomes in patients with STEMI; those that have, have shown it to be a predictor of death and heart failure [8].

The aims of the current analysis were to: (1) define the change in LVEDP over time after reperfusion of the infarct artery, and (2) assess the prognostic value of the elevated LVEDP in STEMI patients. To achieve these aims, we utilized data from the Thrombolysis in Myocardial Infarction (TIMI) II (ClinicalTrials.gov Identifier: NCT00000505) study [9].

## Methods

**Fig. 1:**
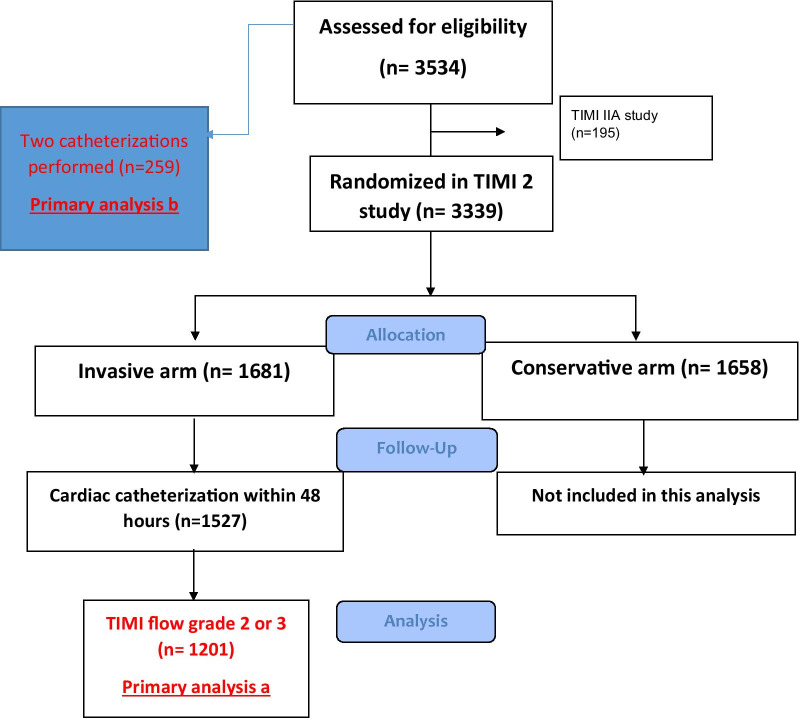
Flow chart demonstrating recruiting to Thrombolysis in Myocardial Infarction (TIMI) II study and the current analysis

TIMI II was a multicentre, randomized clinical study that compared two management strategies for patients with STEMI treated with thrombolytic therapy. The trial recruitment commenced in June 1984. According to the protocol, STEMI patients presenting within 4 h of symptom onset received a 6-h infusion of rt-PA along with aspirin and heparin and were then randomised to an invasive or conservative treatment arm. The invasive strategy involved cardiac catheterization within 18 to 48 h of randomization followed by coronary revascularization by means of percutaneous transluminal coronary angioplasty or coronary artery bypass grafting when appropriate. The patients in the conservative treatment arm of the TIMI II study had cardiac catheterization and revascularization only when prompted by episodes of spontaneous or provoked ischemia at exercise stress test, therefore we excluded them from this analysis. A total of 3534 STEMI patients were assessed for eligibility and 3339 patients were randomized to either an invasive (n = 1681) or a conservative (n = 1658) strategy after intravenous rt-PA. To make our cohort as relevant as possible to modern pharmaco-invasively managed cohorts, only the patients in the invasive arm of TIMI II study who had patent infarct-related artery, as defined by TIMI flow grade 2 and 3 (n = 1201) at the time of first catheterization, demonstrating successful reperfusion with fibrinolysis, are included in the current analysis (Fig. 1). Upon recruitment, a total of 400 out of 3534 patients were planned to undergo second cardiac catheterization prior to hospital discharge to assess change in LVEDP, left ventricular volumes and systolic function. Subsequently, 259 patients with TIMI flow grade ≥ 2 eventually underwent a second (pre-hospital discharge) catheterization. All patients received aspirin, heparin and beta-blockers. The LVEF and LVEDP were measured during left heart catheterization via a pigtail catheter before coronary angiography [9, 10].

The TIMI II study data was accessed through Biologic specimen and data repository information coordinating centre (BioLINCC), which is an initiative of National Heart Lung and Blood Institute of the National Institutes of Health [11]. The current analysis was approved by the Hunter New England Local Health District Ethics Committee, the Human Research Ethics Committee at our institution.

For the current analysis, the whole cohort of 1201 patients was divided into 4 quartiles based on the LVEDP. The primary end points in the present analysis were: (a) change in LVEDP in 259 patients with TIMI flow grade ≥ 2 undergoing a second (pre-discharge) catheterization, and (b) all-cause mortality and heart failure admissions, comparing different quartiles of LVEDP for the whole cohort of 1201 patients. The secondary end points included all-cause mortality and heart failure admissions, comparing different quartiles of LVEDP in patients with preserved ejection fraction (i.e. LVEF ≥ 40%), and correlation between LVEDP and LVEF for the whole cohort of 1201 patients. The LVEF was examined first as a continuous outcome variable and then dichotomised at > 40%. The American college of Cardiology/American heart association definition of left ventricular systolic dysfunction was used for the current analysis with mild dysfunction defined as LVEF 40–49%, moderate dysfunction as LVEF 30–39% and severe dysfunction as LVEF < 30% [12].

We used the Shapiro–Wilk test to assess normality of distribution of the data. Continuous parametric variables are presented as the mean ± standard deviation and compared using t-test. Non-parametric variables are presented as median and interquartile range (IQR). Categorical variables are presented as number and percent and compared using Chi square test. Kaplan–Meier methods were used to estimate event rates at follow-up and to plot time-to-event curves; comparisons were made using the log-rank test. Pearson’s correlation coefficient was used to analyse correlation between LVEF and LVEDP. Univariate and multivariate cox regressions were used to model the outcomes of death and heart failure using LVEDP as categorical variable and LVEDP quartiles.

All statistical analyses were programmed using STATA and SAS v9.4 (SAS Institute, Cary, North Carolina, USA).

## Results

The baseline characteristics of the 4 quartiles for the whole cohort are described in Table 1. The mean and median LVEDP for the whole cohort were 18 ± 8 mmHg and 18 mmHg (IQR: 12–23) respectively. A total of 259 patients (Mean age 56 ± 10 years) had successful pre-hospital discharge (PHD) cardiac catheterization performed. Their baseline characteristics are described in Table 2. The PHD catheterization occurred with a delay of 4 ± 2 days from the baseline catheterization. The median LVEDP decreased from 18 mmHg (1QR: 12–22) to 15 mmHg (IQR: 10–20) from the first to the PHD catheterization (p = 0.01). There was no difference in left ventricular systolic function or volumes from first to PHD catheterization. (Table 3).

**Table 1:** Baseline characteristics of the 4 quartiles

Variables	Quartile 1 (N = 334)	Quartile 2 (N = 336)	Quartile 3 (N = 258)	Quartile 4 (N = 273)	*P* value
Age (years)—mean (SD)	57 (10)	56 (10)	56 (10)	58 (10)	0.7
Male-N (%)	265 (79)	278 (83)	223 (86)	226 (83)	0.6
Smoking—N (%)	259 (78)	273 (81)	207 (80)	195 (71)	**0.04**
DM—N (%)	25 (7)	39 (12)	32 (12)	49 (18)	**0.04**
HTN—N (%)	119 (36)	125 (37)	87 (34)	122 (45)	**0.05**
Creatinine (mg/dl)—mean (SD)	1.1 (0.3)	1.1 (0.3)	1.1 (0.2)	1.2 (0.3)	0.8
Platelets (per ml)—mean (SD)	289 (83)	282 (62)	281 (81)	288 (83)	0.9
Haemoglobin (mg/dl)—mean (SD)	14.8 (1.4)	14.9 (1.3)	15 (1.4)	15 (1.4)	0.8
Anterior MI—N (%)	161 (48)	157 (47)	131 (51)	164 (60)	**< 0.001**
Ejection fraction (%)—Median (IQR)	51 (44–59)	50 (42–57)	47 (39–54)	45 (35–51)	**0.001**
End-diastolic volume (ml)—median (IQR)	112 (91–139)	124 (104 –149)	129 (102–160)	129 (104–159)	**< 0.001**
End-systolic volume (ml)—median (IQR)	54 (41–71)	63 (48–79)	68 (50–89)	72 (53–90)	**< 0.001**
Cardiac output (ml)—median (IQR)	4.4 (3.4–6)	4.5 (3.6–5.9)	4.6 (3.5–6.1)	4.4 (3.1–5.9)	0.5

*SD* standard deviation, *DM* diabetes mellitus, *HTN* hypertension, *MI* myocardial infarction, *N* number, *IQR* interquartile range

**Table 2:** Baseline characteristics of patients undergoing pre-hospital discharge catheterization

Patients undergoing pre-hospital discharge catheterization (N = 259)	
Age (years)—N (%)	56 (10)
Males—N (%)	266 (86)
HTN—N (%)	114 (37)
DM—N (%)	40 (13)
Smoking—N (%)	244 (78)
Haemoglobin—mean (SD)	14.8 (1.4)
Creatinine—mean (SD)	1.1 (0.3)
Platelets—mean (SD)	273 (71)

*DM* diabetes mellitus, *HTN* hypertension, *N* number, *SD* standard deviation

**Table 3:** Change in LVEDP, LVEF and left ventricular volumes from 1st to pre-hospital discharge catheterization

	1st catheterization	Hospital discharge catheterization	*P* value
LVEDP (mmHg)—median (IQR)	18 (12–22)	15 (10–20)	**0.001**
LVEDV (ml)—median (IQR)	133 (109–164)	143 (113–174)	0.12
LVESV (ml)—median (IQR)	63 (49–89)	72 (50–97)	0.2
LVEF (%)—median (IQR)	50 (42—58)	49 (42–57)	0.6
CO (L/min)—median (IQR)	5.1 (4–6.4)	4.8 (3.9–5.8)	0.1
Stroke volume (ml)—median (IQR)	64 (49–79)	66 (52–83)	0.4

*LVEDV* left ventricular end-diastolic volume, *LVESV* left ventricular end-systolic volume, *LVEF* left ventricular ejection fraction, *CO* cardiac output, *IQR* interquartile range, *LVEDP* left ventricular end-diastolic pressure

**Fig. 2:**
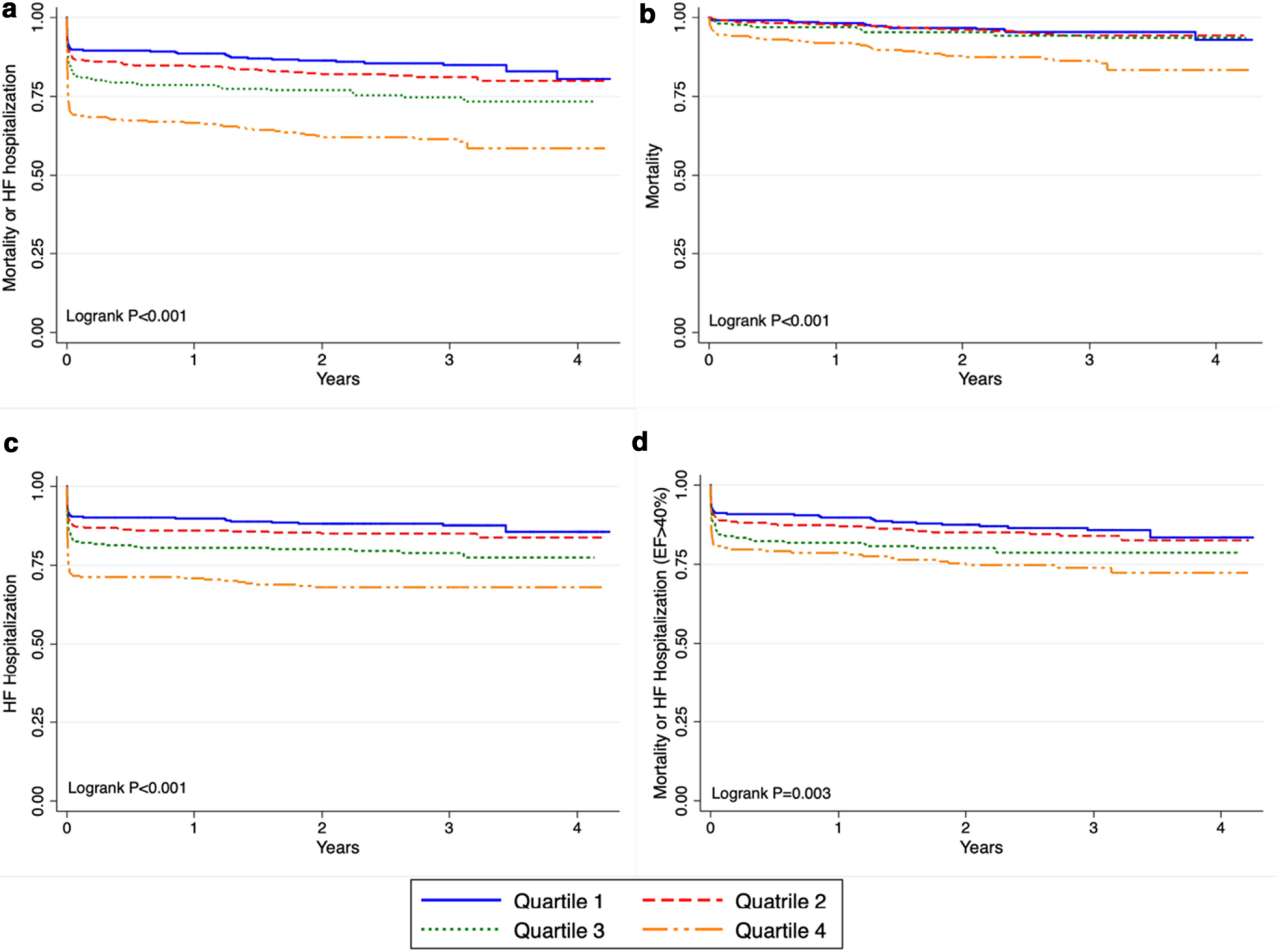
All-cause mortality and heart failure admissions for the whole cohort and for patients with left ventricular ejection fraction ≥ 40%. Outcomes for whole cohort and for patients with LVEF > 40%

**Fig. 3:**
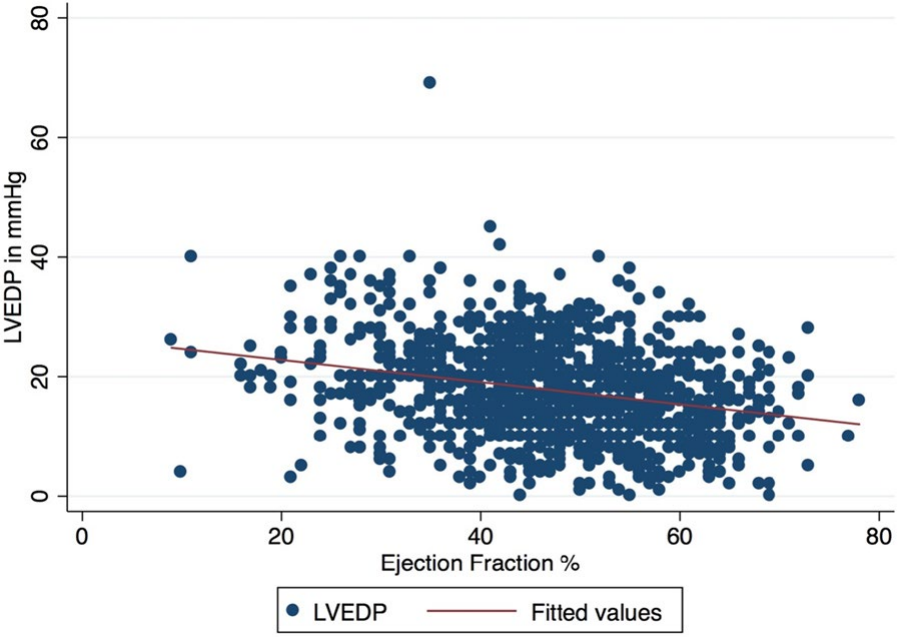
Correlation between left ventricular ejection fraction and left ventricular end-diastolic pressure. For 2% decrease in LVEF, there was 10 mmHg rise in LVEDP (R^2^ = 0.07, *P* < 0.01). Relationship between LVEDP and LVEF. *LVEDP* left ventricular end-diastolic pressure, *LVEF *left ventricular ejection fraction

During a median follow up of 3 (IQR: 2.1–3.2) years, quartile 4 (highest LVEDP) had the highest incidence of mortality and heart failure admissions. (Table 4, Fig. 2a–c) There was a weak inverse correlation between LVEDP and LVEF (Fig. 3). For each 10-mmHg increase in LVEDP, there was 2% decrease in LVEF (95% CI − 0.22 to − 0.14, *P* =  < 0.01, R^2^ = 0.07). In multivariable regression analyses, age, diabetes mellitus, anterior STEMI, elevated LVEDP and reduced LVEF were predictors of death or heart failure. (Table 5).

**Table 4:** LVEDP Quartiles and all-cause mortality and heart failure

	Quartile 1	Quartile 2	Quartile 3	Quartile 4	*P* value
N	334	336	258	273	
LVEDP (mmHg)—median (IQR)	10 (7–12)	16 (15–17)	20 (20–22)	27 (25–30)	**< 0.001**
All-cause mortality—n (%)	15 (5)	17 (5)	15 (6)	39 (14)	**< 0.001**
Heart failure—n (%)	41 (12)	51 (15)	54 (21)	86 (32)	**< 0.001**

*N* number, *LVEDP *left ventricular end-diastolic pressure

**Table 5:** Predictors of death or heart failure

Outcome	HF or death using LVEDP as categorical variable			
	Univariate analysis		Multivariate analysis	
Predictors	Odd ratio	95% CI (*P* value)	Odd ratio	95% CI (*P* value)
(a) LVEDP as categorical variable				
Age	1.05	1.03–1.06 (< 0.001)	1.06	1.04–1.08 **(< 0.001**)
Female	1.89	1.36–2.6 (< 0.001)	1.4	0.93–2.13 (0.1)
Anterior MI	2.31	1.75–3.06 (< 0.001)	2.02	1.43–2.84 (**< 0.001**)
HTN	1.32	1.01–1.73 (0.04)	0.98	0.69–1.39 (0.9)
DM	2.38	1.65–3.42 (< 0.001)	1.84	1.17–2.88 (**0.008**)
CKD	1.64	1.23–2.18 (0.001)	1.27	0.89–1.83 (0.2)
LVEF	0.93	0.92–0.94 (< 0.001)	0.94	0.9–0.98 (**0.003**)
LVEDV	1.002	0.99–1.006 (0.07)		
LVESV	1.01	1.01–1.02 (< 0.001)	0.99	0.97–1.02 (0.9)
LVEDP ≥ 18 mmHg	2.4	1.8–3.2 (< 0.001)	1.7	1.2–2.4 (0.002)

Outcome	HF or death using LVEDP quartiles			
	Univariate analysis		Multivariate analysis	
Predictors	Hazard ratio	95% CI (*P* value)	Hazard ratio	95% CI (*P* value)
(b) LVEDP quartiles				
Age	1.04	1.02–1.05 (< 0.001)	**1.04**	**1.03–1.05 (< 0.001)**
Female	1.7	1.3–2.2 (< 0.001)	**1.4**	**1.01–1.9 (0.04)**
Anterior MI	2.1	1.6–2/7 (< 0.001)	**1.7**	**1.3–2.2 (< 0.001)**
HTN	1.2	0.9–1.6 (0.072)	0.9	0.7–1.2 (0.54)
DM	2	1.5–2.7 (< 0.001)	**1.5**	**1.1–2.1 (0.01)**
CKD	1.5	1.2–1.9 (0.001)	1.2	0.9–1.6 (0.21)
LVEF	0.94	0.93–0.95 (< 0.001)	**0.95**	**0.93–0.98 (0.001)**
LVEDV	1	0.9–1.01 (0.089)	1	0.9–1.01 (0.45)
LVESV	1.008	1.006–1.01 (< 0.001)	0.99	0.98–1.01 (0.85)
LVEDP Quartiles (Comparison with quartile 1)				
Quartile 2	1.3	0.9–1.8 (0.21)	1.2	0.8–1.8 (0.43)
Quartile 3	1.7	1.2–2.5 (0.003)	1.4	0.9–2.1 (0.12)
Quartile 4	3	2.1–4.2 (< 0.001)	1.9	**1.3–2.8 (0.001)**

*DM* diabetes mellitus, *HTN* hypertension, *MI* myocardial infarction, *CI* confidence interval, *CKD* chronic kidney disease, *LVEF* left ventricular ejection fraction, *LVEDV* left ventricular end-diastolic volume, *LVESV* left ventricular end systolic volume, *LVEDP* left ventricular end-diastolic pressure

To determine whether the relationship between LVEDP and outcomes persisted in those with preserved or mildly reduced systolic function, we performed a secondary analysis excluding those with moderate to severe reduction in LVEF. In patients with LVEF > 40% (normal or mild systolic dysfunction), (n = 933, mean age = 56 ± 10 years), the median LVEDP was 16 mmHg (IQR: 12–24 mmHg). Quartiles 1 and 4 had median LVEDP of 10 mmHg (IQR: 7–11 mmHg) and 26 mmHg (IQR: 24–30 mmHg) respectively. During a median follow up of 3 years (IQR: 2.1–3.3 years) we found a similar relationship between LVEDP and outcomes, with the incidence of all-cause mortality and heart failure admissions being highest in quartile 4 (HR 1.9 (95% CI 1.3–2.8, p = 0.001) (Table 6, Fig. 2d).

**Table 6:** LVEDP Quartiles and outcomes in patients with LVEF > 40%

	Quartile 1	Quartile 2	Quartile 3	Quartile 4	*P* value
N = 933	284	209	232	208	
LVEDP (mmHg)—median (IQR)	10 (7–11)	15 (14–16)	20 (18–21)	26 (24–30)	**< 0.001**
All-cause mortality—N (%)	10 (4)	11 (5)	11 (5)	20 (10)	**0.03**
Heart failure—N (%)	32 (11)	20 (10)	42 (18)	42 n	**0.003**

*N* number, *IQR* interquartile range, *LVEDP* left ventricular end-diastolic pressure

## Discussion

Our study has several important findings. First, we define the natural history of LVEDP following reperfused STEMI for the first time. There is a very small reduction in LVEDP over the first few days after reperfusion, but this is of uncertain clinical significance. Second, STEMI patients with elevated LVEDP have higher rates of mortality and heart failure admissions. Third, even in patients with preserved LVEF or mild systolic dysfunction (i.e. LVEF > 40%) following STEMI, LVEDP remained a significant predictor of heart failure admissions and death. Fourth, the correlation between LVEDP and LVEF in STEMI patients, although statistically significant, was quite modest. Taken together, these findings suggest LVEDP could more accurately risk stratify patients after reperfused STEMI.

The management of STEMI has evolved over the decades since the TIMI II study. All patients in this trial had aspirin, heparin, rt-PA, beta blockers, nitrates and frusemide only. The guideline recommended STEMI management in the current era is either primary percutaneous coronary intervention (PCI) or early fibrinolysis followed by rescue or routine PCI [13]. The use of drug eluting stents for PCI, statins and dual anti-platelet therapy have further improved the prognosis of these patients. Similarly, availability of angiotensin converting enzyme inhibitors, angiotensin receptor blockers and mineralocorticoid antagonists have changed the dynamics of post myocardial infarction adverse remodelling and left ventricular dysfunction [14]. We restricted our analysis to the patients who had successful reperfusion with an open infarct-related artery i.e. TIMI flow grade 2 and 3 to ensure our analysis is as relevant as possible to the current clinical practice of early revascularisation.

The proposed mechanism of elevated LVEDP resulting in poor outcomes is adverse left ventricular remodelling (LVR) with subsequent left ventricular fibrosis (± dilatation), development of heart failure and scar-related ventricular arrhythmias. As per the Law of Laplace (left ventricular wall stress = (LVEDP x radius)/ (2 × wall thickness), the LVEDP contributes to wall stress, the primary driver of LVR following STEMI [15]. Thus, theoretically, interventions to reduce LVEDP, may in turn, reduce post-infarction remodelling, heart failure and mortality.

Our study adds to the existing literature showing that hemodynamic assessment provides important prognostic information in patients with STEMI. In the current era, several studies have evaluated the prognostic value of measuring LVEDP at primary PCI. Bagai et al. found in their cohort of 1909 patients undergoing primary PCI that the 90-day mortality after STEMI was higher (4.1% vs. 2.2%; P = 0.007) in patients who had an LVEDP above the median (> 22 mm Hg) [16]. Similarly, Planer et al. showed that LVEDP was an independent determinant of adverse outcomes in multivariate analysis of 2797 patients undergoing primary PCI in the HORIZONS-AMI trial. Patients with LVEDP > 18 mm Hg (above the median) had increased risk of death at 30 days (hazard ratios 2.0; 95% confidence interval 1.20–3.33; P = 0.007) and 2 years (hazard ratios 1.57; 95% confidence interval 1.1 to 2.2; P = 0.009) compared to patients with LVEDP < 18 mmHg [17]. It is interesting to note that the median LVEDP in our cohort was the same as in the HORIZONS-AMI trial, suggesting modern medical therapy has done little to change the LVEDP acutely after STEMI.

A few hemodynamic risk stratification models of patients with STEMI have been proposed and LVEDP, when used in conjunction with other hemodynamic parameters, and left ventricular systolic function have demonstrated prognostic capacity in these models. Sola et al., in their single centre retrospective analysis of 219 STEMI patients, showed that a systolic blood pressure to LVEDP ratio ≤ 4 identified the group of STEMI patients at high risk of in-hospital death [18]. Similarly, in an analysis of 1283 STEMI patients, Ndrepepa et al. demonstrated that a lower LVEF/LVEDP ratio was independently associated with increased risk of cardiac mortality up to 8 years after primary PCI [19]. The LVEF/LVEDP ratio, but not LVEF or LVEDP alone, improved predictive accuracy of multivariable models with respect to long-term cardiac mortality.

Describing the natural history of LVEDP at baseline followed by a pre-discharge repeat cardiac catheterization is an interesting and unique finding of this study. It is interesting to note that despite successful reperfusion (TIMI flow grade ≥ 2) in this cohort, only a small proportion of patients had a significant decline in LVEDP from baseline to PHD catheterization. Thus, pharmacological and/or mechanical therapies to achieve early reduction of LVEDP may reduce subsequent heart failure and mortality.

Despite having adequate evidence-based anti-heart failure medications, post-myocardial infarction heart failure is still a clinical concern even when achieving the guideline-recommended door to balloon times in the contemporary management of STEMI in the developed world [20, 21]. This is related to ischemia and left ventricular loading resulting in adverse remodelling. Recently, there has been renewed interest in the early unloading of the left ventricle in the STEMI patients [22, 23]. The LVEDP, along with left ventricular end-systolic pressure, left ventricular volume and heart rate, are the drivers behind left ventricular loading and increased oxygen demand. This left ventricular loading correlates with the magnitude of myocardial injury in the STEMI patients and affects clinical outcomes. Satıroğlu et al. studied the acute impact of opening the infarct related artery in STEMI on left ventricular hemodynamic changes and compliance [24]. A total of 29 patients with anterior and inferior STEMI had aortic pressure and LVEDP measured before and after primary PCI in the cardiac catheterization laboratories. After successful reperfusion, the left ventricle compliance improved and LVEDP decreased by 6 ± 3 mmHg (p = 0.0005) and 5 ± 6 mmHg (*P* = 0.026) in inferior and anterior STEMI, respectively. This shows that opening the infarct related artery not only relieves ischemia but also has beneficial acute hemodynamic effects. Our study adds to the existing knowledge, showing that LVEDP continues to fall modestly over the next few days following reperfusion in STEMI.

Current guidelines focus on routine measurement of LVEF post myocardial infarction, due to its ease of measurement both at baseline and during follow up. Non-invasive parameters of diastolic function involving echocardiography have only a modest correlation with invasive LVEDP measurement [25]. Our study suggests that measuring LVEDP provides additional prognostic information in STEMI patients.

Despite the advancements in the management of patients with STEMI, elevated LVEDP has seldom been used as a treatment target. The elevated LVEDP in STEMI patients without cardiogenic shock can be safely reduced pharmacologically in the acute setting after primary PCI with a combination of nitrates and diuretics [26]. Thus, it is enticing to speculate that the early reduction in LVEDP in STEMI patients, specifically targeting those with the highest LVEDP, will reduce LVR and improve outcomes; however this hypothesis remains to be tested in prospective randomised controlled trials, such as the ongoing Reduction of End Diastolic Pressure in Acute Myocardial Infarction [REDPAMI] trial (registered at ANZCTR.org.au; registration number ACTRN12618000096257).

Our study has some important limitations. The TIMI II study recruited patients over 30 years ago and this is a post hoc analysis only. Therefore, it is subject to all the limitations of post-hoc analyses; however, we should point out that this relationship was hypothesised a priori and was not a result of data mining in this dataset. In recent decades, the treatment of STEMI has revolutionary changes, including primary PCI, dual antiplatelet therapy with potent P2Y12 inhibitors, routine use of angiotensin converting enzyme inhibitors (or angiotensin receptor blockers) and mineralocorticoid antagonist in left ventricular dysfunction and statins for dyslipidmia. Although the results from the TIMI II study could not reflect the current STEMI management it does highlight the natural history of elevated LVEDP and its effect on post myocardial infarction heart failure and mortality. It adds to our understanding the pathophysiology and can guide the future treatments, as improving post myocardial infarction heart failure outcomes is a big challenge for modern cardiovascular practice [27]. Similarly, the presence of proteinuria in diabetics is an independent predictor of adverse cardiovascular outcomes [28]. Adiponectin and insulin resistance are related to progression of coronary artery disease even in patients with normal glucose tolerance [29]. We did not have that data available. Thus, this analysis should be taken as hypothesis generating, and prospective randomized studies in this field are warranted especially as primary PCI outcomes have plateaued and we are now seeing an increase in survivors who develop heart failure post myocardial infarction.

## Conclusion

In conclusion, following reperfused STEMI, there is only a modest in-hospital drop in LVEDP. Elevated LVEDP is a predictor of death and heart failure hospitalization in patients with reperfused STEMI, even when LVEF is normal/mildly reduced. Future studies are needed to assess the effect of early reduction in LVEDP on the clinical outcomes.

## Data Availability

The datasets analysed during the current study are not publicly available due institutional ethics approval restrictions.

## Abbreviations

TIMI: Thrombolysis in Myocardial Infarction
LVEDP: Left ventricular end-diastolic pressure
STEMI: ST-segment elevation myocardial infarction
LVEF: Left ventricular ejection fraction
IQR: Interquartile range
PHD: Pre-hospital discharge
PCI: Percutaneous coronary intervention
LVR: Left ventricular remodelling
